# Introducing the Mesh Integration (MINT) Index: a standardised ratio scale for assessing in vivo hernia mesh performance

**DOI:** 10.1007/s00464-025-12098-1

**Published:** 2025-09-02

**Authors:** Edward Young, Alex Karatassas, Jean Wong, Peter J. Hewett, Sarah Jesse, Guy J. Maddern

**Affiliations:** 1 Discipline of Surgery, Faculty of Health and Medical Sciences, The University of Adelaide, PO Box 328, Torrensville, South Australia 5031 Australia; 2 Department of Surgery, The Queen Elizabeth Hospital, Woodville South, South Australia Australia; 3 Basil Hetzel Institute, The Queen Elizabeth Hospital, Woodville South, South Australia Australia

**Keywords:** Hernia, Mesh, Tissue integration, Index

## Abstract

**Background:**

One in 15 people throughout their lifetime will develop an incisional hernia. Poor mesh tissue integration is thought to be responsible for hernia repair failure years after surgery. The aim was to develop a mesh integration index for standardised assessment of in vivo hernia mesh behaviour in the abdominal wall.

**Methods:**

The core properties of the Index were defined by the authors a priori, requiring the Index to be objective, reproducible, independent of animal models, compatible with past and future research and utilises equipment typically available at biomedical research institutes. The structure of the Index was built upon experience obtained in an earlier pilot study, incorporating key measurements and testing methods identified from previous publications. High precision test methods aligning with local and international standards were utilised where possible.

**Results:**

The proposed Mesh Integration (MINT) Index is a 0–5 ratio scale that numerically represents the integration, fibrosis, adhesion and degradation behaviour of hernia mesh in vivo, using pre-existing standardised assessments. Assessments fell into four broad categories of visual, histological, biomechanical and molecular. Biomarkers were not included due to uncertainty of interpretation. Score calculations and rationale were explained in detail.

**Conclusion:**

An objective index was created to assess in vivo hernia mesh behaviour. The index will need validation via studies using explanted mesh tissue complex from living abdominal walls.

**Supplementary Information:**

The online version contains supplementary material available at 10.1007/s00464-025-12098-1.

Incisional hernias are a complication of abdominal surgery. One in fifteen people throughout their lifetime will develop an incisional hernia [1, 2]. Even though there are preventative measures [3], developing incisional hernias following major abdominal surgery continues to remain a material risk.

The current gold standard of incisional hernia repair is reduction of hernia sac, tension-free suture closure of hernia defect, and abdominal wall reinforcement via mesh insertion [4], Although mesh is superior to suture-only closures, 50% of incisional hernia mesh repairs are still expected to fail within 5-year post-repair [4]. Whilst early mechanical stability of mesh tissue interface is critical to foster tissue regeneration [5], poor mesh tissue integration is postulated to be primarily responsible for repair failures and hernia recurrences observed years after surgery [6].

Mesh tissue integration describes the observed phenomenon of tissue ingrowth when mesh is implanted. An inflammatory response is initiated that precipitates both tissue healing and foreign body response [7]. New cells will attempt to infiltrate the mesh, while fibrotic tissue will be deposited on any exposed mesh surface. Finding a ‘sweet spot’ between integration and fibrosis is critical for long-term mesh repair stability [6]. If effective porosity of mesh is too small, a bridging effect by fibrous tissue may occur and prevent ingrowth of tissue [8]. A limited degree of mesh tissue integration performance may be inferred from its physical structure and properties, though products may not necessarily have identical performance under normal and pathological conditions. Yet this is often assumed in clinical practice out of convenience, due to lack of clear guidance. There is an urgent need for an evidence-based approach to quantify mesh tissue integration, and allow surgeons to tailor mesh selection to patient circumstances in an objective fashion.

A major limitation of existing studies examining mesh tissue integration is methodology variation and difficulty of comparing results [9]. With over 150 hernia mesh products actively registered on the United States Food and Drug Administration (FDA) database [10], an index system with a standardised methodology is perhaps a better approach.

The aim of this study was to develop a mesh integration index for standardised assessment of in vivo hernia mesh behaviour in the abdominal wall.

## Materials and methods

The core properties of the Index were predefined by the authors a priori, requiring the Index to be objective, reproducible, independent of animal models, compatible with past research, future-proof, utilise equipment typically available at biomedical research institutes, and have an intuitive scale for easy interpretation and clinical usage.

Reference was made to past works by Karatassas et al. and Patiniott et al. [6, 11]. In an extended commentary, Karatassas et al. envisioned a 1 to 5 ordinal scale to classify in vivo mesh behaviour, based on objective test findings, such as histological analysis, biomechanical testing and biological marker levels. While the potential utility of the Index was well described, structure of the index and how to convert test results to the Index was absent.

Patiniott et al. attempted to establish the index by directly utilising standardised scoring systems for histological and adhesional changes, along with biomechanical test results of the mesh tissue interface in a porcine model [11]. Though the porcine study was short in duration and small in numbers, it demonstrated the utility of standardised scoring systems over time in recording parameters associated with the mesh tissue interface. More importantly, the data gathered by Patiniott et al. highlights several barriers to translating objective test results to clinical utility. An ordinal scale with five steps is likely too imprecise to describe the changes at the mesh tissue interface, as evident by the near-uniformly reported fibrosis scores of 4 at weeks 2 and 4. Ordinal scales additionally have inherent limitations during statistical analyses, and a ratio scale is more comprehensive when available. Directly using raw scores from a single scoring system in the Index reduces compatibility if the same system is not utilised. The invasive nature of examining the mesh tissue interface limits the assessment to animal models or mesh explants in humans. Prospective clinical trials is not possible due to significant abdominal wall disruption. The overall results were notably difficult to summarise in the absence of a uniform system. For results to be reproducible and usable by others, a clear explanation needs to be present as to how test scores are converted into the Index score.

To address these identified problems, a scoping review of the literature was undertaken by the authors in January 2024 in accordance to Preferred Reporting Items for Systematic Reviews and Meta-analysis Extension for Scoping Reviews (PRIMSA-ScR) Guidelines, using a pre-defined protocol [paper submitted but not yet accepted for publication]. Briefly, databases of PubMed and Scopus were searched, using keyword combinations and derivatives of ‘hernia’, ‘mesh’, ‘integration’ and ‘incorporation’. An independent search of the citation list and review of the grey literature was also performed. All clinical papers describing animal studies investigating abdominal wall mesh tissue integration were included, with exclusion of non-English papers, human studies, reviews, commentary and letters to editors. A total of 80 relevant papers were identified from 1,107 abstracts, and data were extracted using a predefined extraction template. The literature review did not reveal any pre-existing index system that would satisfy the design requirements outlined earlier. Numerous similar but distinct scoring systems assessing histology, adhesions or integration were identified, such as Modified Hopkins Score and Modified Diamond Scale [12, 13]. Method of grading included categorical systems using grading sheets, and numerical systems through direct measuring.

Using the pilot study as a starting point, assessments identified in the scoping review with common themes were grouped. Grouping was necessary as authors of various studies often used different tools to assess the same item at the mesh tissue interface [14]. For standardised scoring systems, questions from different systems were combined if they were identical or of sufficient similarity. If they were different, then the questions were kept as separate items. This allowed inclusion of all commonly utilised validated scoring systems, providing forward and backward compatibility when analysing existing and future research data. For laboratory tests, such as biomechanical testing, methodology of testing were grouped. Recognising that there are likely variations in testing methods, devices and setup, reference to local and international standards were made to streamline methodology (Table 1). Where such uniform methodology is not in existence, the most accurate and relatively simple method to perform in a standard laboratory setting was chosen. When possible, the more precise method of assessment was utilised, e.g., measuring actual area of integration using computerised program, as opposed to a visual percentage-based scoring system.

**Table 1:** National and international standards referenced

Standards / guidelines	Name
ISO 10993–6:2016	Biological Evaluation of Medical Devices—Part 6: Tests for local effects after implantation
ISO 10993–13:2010	Biological Evaluation of Medical Devices—Part 13: Identification and quantification of degradation products from polymeric medical devices
ATSM F2255-05	Standard Test Method for Strength Properties of Tissue Adhesives in Lap-shear by Tension Loading
ATSM D4850-13	Standard Terminology Relating to Fabrics and Fabric Test Methods

*ISO* International Organization for Standardization, *ASTM* American Society for Testing and Materials international standards

To ensure the Index remained independent of animal models, the Index and testing methods were setup without using in vivo data, such as animal studies. Restricting the Index to a specific animal model or species could potentially introduce bias, and limits utility as not all laboratories may have the exact same animal species and living conditions. The downside to this design is that the output of the Index is open for interpretation, and does not provide guidance on what the optimal value for the Index is. As value of each testing method remained unknown, equal weighting was given to all methods. All included test methods were required to have a meaningful minimum and maximum, in order to align with the Index values as previously defined. Test methods without a meaningful minimum or maximum were excluded. Score values were converted to a percentage to facilitate calculations. All conversions and formulas were to be displayed and explained clearly such that it may be reproducible.

This study was not preregistered due to its theoretical nature, though it used an a priori approach by first defining the key targets the Index must meet. The scoping review referenced here used a predefined protocol and search strategy (to be published separately), but was not eligible for registration with PROSPERO.

## Results

The proposed Mesh Integration Index (hereafter referred to as the Index) is a 0–5 ratio scale, rounded to one decimal. Scores are calculated for each individual domain, and divided into five intervals for descriptive purposes (Table 2). Each interval has a distinct name and corresponds to a score range, i.e., minimal for 0 to ≤ 1.0, mild for > 1.0 to ≤ 2.0, moderate for > 2.0 to ≤ 3.0, extensive for > 3.0 to ≤ 4.0 and maximal for > 4.0 to ≤ 5.0. Domain scores are calculated from mean scores of standardised assessments for a single mesh tissue sample at a specific observation time point. The Index itself does not have a global score. If the calculated score falls outside the scale range, they are to be interpreted as 0 or 5, whichever is closest. Comparison of mesh tissue behaviour using the Index should be performed over multiple time points.

**Table 2:** Proposed mesh integration index, and scores

Score	I–Integration	F–Fibrosis	D–Degradation	A–Adhesion
> 4.0 to ≤ 5.0	Maximal	Maximal	Maximal	Maximal
> 3.0 to ≤ 4.0	Extensive	Extensive	Extensive	Extensive
> 2.0 to ≤ 3.0	Moderate	Moderate	Moderate	Moderate
> 1.0 to ≤ 2.0	Mild	Mild	Mild	Mild
0.0 to ≤ 1.0	Minimal	Minimal	Minimal	Minimal

The Index has 4 domains of Integration, Fibrosis, Adhesion and Degradation. These domains were chosen for their immediate relevance to clinical practice. Emerging evidence may modify the domains in the future. Integration and Fibrosis are two key components of biological fixation. Adhesion is relevant to intraperitoneal techniques. Degradation is relevant as polymers used in mesh, previously thought to be ‘inert’, can degrade over time [15].

Each domain has a specific group of standardised assessments and corresponding assessment scores. Assessment scores are calculated from component scores, and converted to percentages by comparing to a control sample or a pre-defined value. The assessment scores fall into four broad categories (Table 3). Assessment scores have equal weighting within a domain. Component scores are unique across domains to prevent collinearity. The domain score is calculated based on the mean of assessment scores, and then multiplied by 5 to plot on the scale.

**Table 3:** Summary of assessment scores utilized to calculate the domain scores of the Index

	Integration	Fibrosis	Degradation	Adhesion
Visual	% visual integration	% visual shrinkage	% visual degradation	% visual adhesion
Histology	% integration histology	% fibrosis histology	% degradation histology	–
Biomechanical	% shear ratio, elastic % shear ratio, ultimate	–	%$\Delta$ mesh tensile strength	–
Molecular	–	–	%$\Delta$ Carboxyl Index % MAD∆absorption	–

*% MAD∆absorption* mean absolute deviation of percentage change in spectra peaks’ absorption values

$$Total\, Domain\, Score=5 \times \sum (Visual+Histological+Biomechanical+Molecular)$$

Components or assessments without scores are ignored during calculations. This allows the Index to have a degree of tolerance of missing data, though interpretation of results should be made cautiously.

### Assessment score category—visual

Visual assessment is direct observation of changes and parallels with clinical practice. All items of interest should be photographed or recorded so that repeat assessments can be performed.

For Integration:

Area of integration is defined as an area where mesh is not easily visible, does not lie separate from tissue, and there is visible evidence of tissue ingrowth within pores.

$$\%\, visual\, integration= \frac{area\, of\, integration}{area\, of\, mesh\, post\, implantation}\times 100\%$$

Shrinkage is taken into account using the area of the mesh post-implantation. The value of visual integration is between 0 and 100%.

For Fibrosis:

The area before and after implantation is compared to assess shrinkage.

$$\%\, visual\, shrinkage=\frac{(area\, of\, mesh\, pre\, implantation\, -\, area\, of\, mesh\, post\, implantation) }{area\, of\, mesh\, pre\, implantation}\times 100\%$$

A negative value indicates the mesh has expanded. There is no mathematical limit to the minimum score, but it is likely no more than -100%, which occurs when the area post-implantation doubles. Maximum score is 100%.

For Degradation:

No existing visual scoring system could be found in the literature that assessed hernia mesh degradation. The search was broadened to polymer degradation, and a quasi-scoring system existed for preservation of plastic cultural artefacts in museums [16]. Using the terminology described in the paper, a new visual degradation scoring system was created (Supplementary 1). The scoring system is qualitative, and only scores whether damage is observed or not. It does not quantify how much damage is present, nor cause of observed damage.

$$\%\, visual\, degradation= \frac{observer\, rated\, visual\, degradation\, score}{8}\times 100\%$$

A percentage from the total component score (out of 8) is calculated. The minimum assessment score is 0%, and the maximum is 100%.

For Adhesion:

The Mesh Tissue Adhesion (META) Score is used (Supplementary 2). This is an international consensus by leading hernia experts that unifies 15 different adhesion scoring systems [17].

$$\%\, visual\, adhesion= \frac{average\, of\, observer\, rated\, META\, Score}{14}\times 100\%$$

A percentage from the total META score (out of 14) is calculated. The minimum component score is 0%, and the maximum score is 100%.

### Assessment score category—histological

Histology sections are to be stained using haematoxylin and eosin, and be assessed under light microscopy with 40 × magnification, using a 2-page standardised histology scoring worksheet (Supplementary 3).

The histology scoring worksheet was adapted from the combination of International Organization for Standardization (ISO) 10993–6:2016 [18], Keating’s scoring system [19], and Jenkins’ scoring system [20]. Each component score for a specific mesh sample is corrected by subtracting scores from control samples. Control samples are to be retrieved for each layer of implantation in each animal. Total scores are calculated by grouping relevant component scores.

$$corrected\, mesh\, sample\, component\, score= mesh\, sample\, component\, score-control\, sample\, component\, score$$

For Integration:

The difference in the average histological scores of desirable and undesirable findings is calculated. A degree of inflammation is required for wound healing, however, excess inflammation and foreign body response are undesirable.

$$\%\, integration\, histology= \frac{desirable-undesirable}{4}\times 100\%$$

$$desirable= \frac{cellular\, infiltration+neovascularisation+connective\, tissue\, deposition}{3}$$

$$\begin{aligned}&undesirable= \\&\quad  \frac{{\left(\begin{aligned}&polymorphonuclear\, cells+lymphocytes+plasma cells \\&\quad+macrophages+giant cells+necrosis\end{aligned}\right)}}{6}\end{aligned}$$

The individual components have equal weighting. It is possible to have a negative integration histology module score by having minimal integration and excessive inflammatory reaction. Minimum score is -100%, and maximum score is 100%.

For Fibrosis:

The percent value for average score of all findings that suggests fibrosis is obtained. Inflammatory scores are not included to avoid overlapping with integration.

$$\%\, fibrosis\, histology= \frac{fibrosis+fatty\, infiltration+fibrous\, encapsulation+minerlisation}{4\times 4}\times 100\%$$

The greater the value, the more pronounced the fibrosis is. Minimum score is 0%, maximum score is 100%.

For Degradation:

Different from visual degradation, implant degradation in histology scoring examines the cross section of implants, and assesses whether there are any local tissue changes from degradation or degradative products.

$$\%\, degradation\, histology= \frac{implant\, degradation}{1\times 4}\times 100\%$$

Minimum score is 0%, maximum score is 100%.

### Assessment score type—biomechanical assessment

A review of ISO and American Society for Testing and Materials International (ASTM) standards indicates there are currently no standards specific to synthetic surgical mesh. Existing methods of tensile testing in the literature include uniaxial, biaxial or ball burst testing [21]. Although the latter two methods likely better mimic the abdominal wall stresses [22], uniaxial testing is much more accessible on standard tensiometer platforms.

**Fig. 1:**
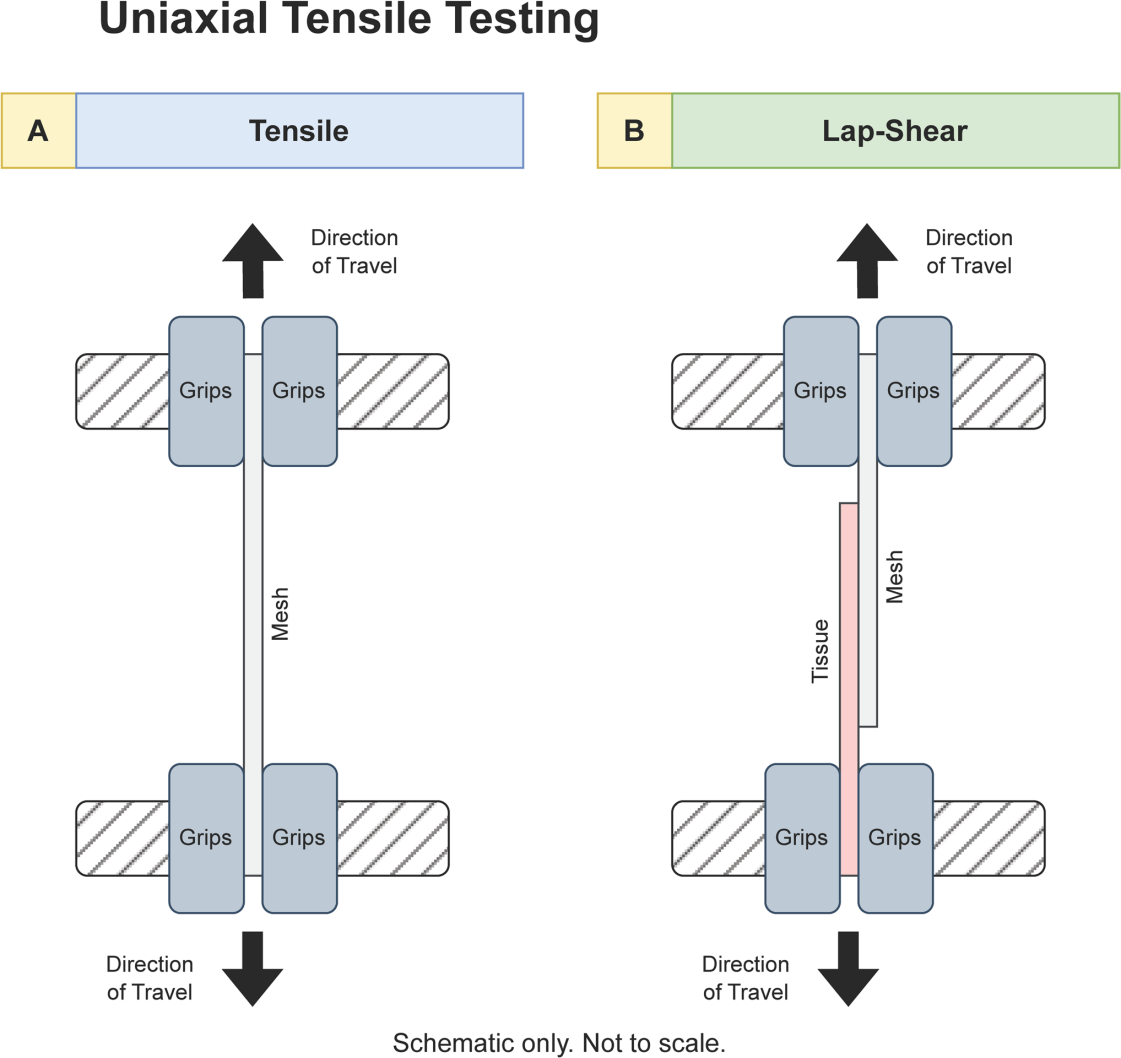
Schematic diagram of uniaxial tensile testing of mesh pieces. Not to scale

For Integration:

The mesh tissue interface may be viewed as the mesh being ‘adherent’ to the underlying tissue [23]. Using ATSM F2255-05 and Yuk et al. as a guide [24, 25], the mesh tissue sample is prepared for gripping (Fig. 1). The width and length of the area of contact between mesh and tissue are recorded. The axis of the sample is aligned to the grips’ central axis of travel, and the velocity of travel is set to 24 mm/min (0.4 mm/s). The data output of Force over Distance is converted to Shear ($\tau$) over Strain ($\varepsilon$) using the following formulas:

$$\tau = \frac{F}{area\, of\, contact}=\frac{F}{width\, of\, specimen \times length\, of\, specimen\, with\, mesh\, in\, contact}$$

$$\varepsilon = \frac{\Delta\, length}{initial\, length\, of\, sample}=\frac{length\, of\, sample\, at\, breakage-initial\, length\, of\, sample}{initial\, length\, of\, sample}$$

The values of interest are shear at the elastic limit (${\tau }_{elastic limit}$), and shear at the ultimate limit (${\tau }_{ultimate limit}$). The ${\tau }_{elastic limit}$ is the maximum shear the interface can sustain while still retaining elastic properties. The ${\tau }_{ultimate limit}$ is the maximal shear the mesh tissue interface can sustain before detachment of mesh occurs.

To standardise these figures, they are compared to the maximal shear stress that can be experienced over 1 cm ^2^ in the human abdominal wall. Maximal intra-abdominal pressure is typically about 225 mmHg (about 30 kPa or 3 N/cm^2^) during intense post-operative vomiting [26]. Assuming an abdominal girth of 100 cm, and thickness of 5 mm (0.5 cm) between the posterior rectus fascia and the peritoneum, then the maximal hoop stress that can be experienced in the abdominal wall is as below [27].

$$radius\, of\, abdomen=\frac{circumference}{2\pi }=\frac{100 cm}{2\pi }\approx 32cm$$

$${\sigma }_{hoop max}=\frac{intra\, abdominal\, pressure \times radius\, of\, abdomen}{thickness\, of\, abdominal\, wall}=\frac{3 N/{cm}^{2} \times 32 cm}{0.5 cm}\approx 48 N/{cm}^{2}$$

Using the larger ${\sigma }_{hoop max}$ as a benchmark, ${\tau }_{elastic limit}$ and ${\tau }_{ultimate limit}$ can be ratioed into a percentage.

$$\%\, shear\, ratio,\, elastic= \frac{{\tau }_{elastic\, limit}}{{\sigma }_{hoop\, max}}\times 100\%= \frac{{\tau }_{elastic\, limit}}{48}\times 100\%$$

$$\%\, shear\, ratio,\, ultimate= \frac{{\tau }_{ultimate\, limit}}{{\sigma }_{hoop\, max}}\times 100\%=\frac{{\tau }_{ultimate\, limit}}{48}\times 100\%$$

The minimum value is 0%. There is no maximum value, but upper limit is likely 100% when ${\tau }_{ultimate\, limit}=$ ${\sigma }_{hoop\, max}$.

For Degradation:

The mesh is to be placed inside the grips of the tensiometer, and aligned to the central axis of grips (Fig. 1). Where applicable, anisotropic meshes are orientated with the warp fibres parallel to the central axis of the grips. The width of the mesh piece is measured. The grips are displaced at a velocity of 24 mm/min (0.4 mm/s).

The force–displacement output is converted to stress–strain. The post-implantation tensile strength of mesh is compared to the pre-implantation strength from the same batch of mesh. This method avoids the need for manufacturer data on mesh tensile strength which may be difficult to obtain, and accommodates any changes that may be introduced as result of processing, such as resterilisation.

$$\%\, \Delta\, mesh\, tensile\, strength= \frac{original\, mesh\, tensile\, strength- ultimate\, mesh\, tensile\, strength}{original\, mesh\, tensile\, strength}\times 100\%$$

The minimum value may be negative, where the tensile properties of the mesh improved with implantation, or was influenced by external factors. The maximum value is 100%, where there is complete failure of tensile strength.

### Assessment score type—molecular

Polymer degradation is a complex process. There is currently no single method of analysis that can provide a comprehensive picture of polymer degradation at both macroscopic and molecular levels [28]. Analytical tools are broadly divided into physical, thermal, chromatography, spectroscopy and respirometry (Table 4) [28].

**Table 4:** Classification of available methods to assess polymer degradation

Type	Aspect of analysis	Analytic method	Available tests
Bulk property changes	Morphology	Physical	Mass loss Light microscopy Scanning / transmission electron microscopy Atomic force microscopy Contact angle Light scattering Mechanical property / tensile testing
	Melting point Crystallinity	Thermal	Thermogravimetric analysis Differential scanning calorimetry Differential thermal analysis
Molecular changes	Molar mass change	Chromatography	Gel permeation chromatography
		Spectroscopy	Nuclear magnetic resonance Secondary ion mass spectroscopy
	Functional groups	Spectroscopy	Fourier’s transformation infrared spectroscopy Ultraviolet visible spectroscopy X-ray photoelectron spectroscopy Fluorescence spectroscopy Nuclear magnetic resonance X-ray diffraction X-ray fluorescence
	Degradation Products	Chromatography	Liquid chromatography mass spectrometry High performance liquid chromatography Gas chromatography mass spectrometry ± Isotope labelling
		Respirometry	Biogas evolution

Adapted from Baidurah [28] and Colachis et al. [29]

Suitability of analytic methods need to consider the samples that are being analysed. Typically hernia mesh is of solid porous nature, relatively large in area but light in weight, has a slow degradative process that is usually concentrated on exposed areas (i.e., surface), not easily soluble, may absorb water, may have biological contamination, and any degradative products that are not attached to the mesh is likely left behind at time of extraction. Tests need to be relatively non-expensive to operate, widely available in standard biomedical laboratory setups, minimal sample preparation, and can efficiently process multiple mesh samples.

Within these requirements, the following analytic methods are likely not suitable: methods confounded by presence of water (mass loss, thermogravimetric analysis, differential scanning calorimetry, differential thermal analysis); methods requiring sample to be soluble in solvent (liquid chromatography mass spectrometry, gas chromatography mass spectrometry, high-performance liquid chromatography, gel permeation chromatography, nuclear magnetic resonance spectroscopy); methods require degradation products to be present (ultraviolet visible spectroscopy, biogas evolution); methods measuring trace elements (X-ray fluorescence); and methods relatively expensive to perform in bulk (X-ray photoelectron spectroscopy, isotope labelling) [28].

Biomedical laboratories are typically equipped with light microscopy and electron microscopy. They are non-destructive, and only minimal sample preparation is required. Likewise, tensile testing to assess mechanical properties are easy to conduct, though it is destructive by nature and does not elicit the reason for change in mechanical properties.

X-ray diffraction assesses crystallinity of polymers by sending X-rays towards the sample and measuring diffraction angles and amount of energy released. As the X-rays need to ‘bounce’ off the sample surface, the porous mesh may present a problem, and destructive sample preparation (e.g., pulverisation) may be required. Additionally, X-ray diffraction device is not typically found in biomedical research facilities, thus this assessment was not included in the current iteration of the Index.

Attenuated Total Reflectance Fourier’s Transformation Infrared Spectroscopy (ATR-FTIR) is a commonly used spectroscopic method of analysing solids and liquids non-destructively. ATR-FTIR is highly sensitive to chemical bond changes and can be used for both identification and quantification. ATR-FTIR has a scanning depth of 1 µm which is adequate for polymer analysis, as degradation reactions from enzymes or agents in physical contact typically occur within 0.01 µm of the surface [29].

For Degradation:

A suitable ATR-FTIR device with a diamond crystal is to be used. The ATR-FTIR is configured to perform 32 scans at 4 cm^−1^ resolution, measuring absorbance between wavenumbers 4000–400 cm^−1^, using Happ-Genzel apodization [30]. A background spectrum is taken first, then a sample is immediately mounted against the crystal window with standardised pressure. The scan is performed at 3 random locations on each mesh sample. The device will automatically subtract the background spectra. The final spectra are baseline-corrected by vertical shifting so that it has a zero value at wavenumber 4000 cm^−1^.

#### Carboxyl index

A carboxyl index (CI) is calculated for polypropylene or polyethylene mesh, using Almond et al.’s area under band criteria [30]. Area under band is calculated using suitable spectra software.

$$Carboxyl\, index\, (CI)= \frac{area\, under\, band\, between\, 1850-1650\, cm^{-1}}{area\, under\, band\, between\, 1550-1420\, cm^{-1}}$$

The CI of post-implant mesh is compared to pre-implant mesh.

$$\%\, \Delta\, Carboxyl\, Index= CI_{post-implant}-CI_{pre-implant}$$

There is no upper or lower limit to $\%\, \Delta\, Carboxyl\, Index$. CI is not applicable to mesh which are made of non-polypropylene, non-polyethylene material, or if the area under bands are superimposed by other substances, such as composite fibres or anti-adhesive coatings.

#### Spectra peak mean absolute deviation

Difficulty of analysing hernia mesh for evidence of degradation is that other unknown substances are likely present, while spectra libraries generally do not have proprietary information on mesh. Additionally, the porous-shaped hernia mesh will result in random variation in how much mesh fibres and surface area is available for infrared radiation to reflect off.

A workaround to these problems is to analyse the overall change in spectra peaks between the test sample and a reference sample. For the same substance, spectral peak positions will be identical. The magnitude of absorption depends on the absorbance properties of the underlying chemical bonds, and has a linear relationship with quantity of chemical bonds (i.e., concentration of substance) as per Beer-Lambert Law. Assuming standard laboratory test conditions, ATR-FTIR scans of the same substance, at the same concentration and with the same thickness will produce near-identical spectra, with slight variations due to background noise [31]. When concentration varies, the spectra between samples will have different absorption values, but identical spectra peaks. The percentage difference in absorption values between sample A and reference sample B at spectra peak M can be expressed as:

$$\begin{aligned}&\%\, \Delta\, absorption\\&\quad = \frac{\left(\begin{aligned}&absorption \, of\, Sample\, A\, at\, peak\, M \\&\quad - absorption\, of\, Reference\, Sample\, B\, at\, peak\, M \end{aligned}\right)}{absorption\, of Reference\, Sample\, B\, at\, peak\, M}\times 100\%\end{aligned}$$

Multiple peaks will produce a group of $\%\Delta absorption$. If two samples had different concentrations of the same chemical, the overall proportion of types of chemical bonds will not change, and we would expect $\%\, \Delta\, absorption$ to be nearly the same between all peaks. If $\%\, \Delta\, absorption$ across multiple peaks are different from each other, then there is an unequal proportion in the type of chemical bonds between the samples, i.e., they are made of different chemicals. The average size of variation of $\%\, \Delta\, absorption$ from its collective mean can be determined by obtaining the $mean\, absolute\, deviation$ (MAD), a statistical concept similar to standard deviation.

$$\%\, MAD\, \Delta\, absorption= \frac{1}{N}\sum_{i=1}^{N}{\left|\% \Delta\, absorption-\%\, mean\, \Delta\, absoprtion\right|}_{i}$$

$$\%\, mean\, \Delta\, absorption= \frac{1}{N}\sum_{i=1}^{N}({\%\, \Delta\, absorption)}_{i}$$

If MAD is zero or near zero for two samples, then there is a consistent change between spectra peaks across the samples, and indicates that the samples are of same composition with varying concentration. The greater the MAD, the more varied differences there are in absorption values of chosen spectra peaks between two samples, indicating different proportions of chemical bond types, i.e., change in chemical structure and degradation. The more peaks are chosen between spectra, the more accurate MAD is at representing the spectra. The minimum value of MAD is 0. There is no maximum value of MAD.

### Assessment score type—biomarkers

Consideration was given to testing for collagen I, collagen III, matrix metalloproteinase (MMP), vascular endothelial growth factor (VEGF) and transforming growth factor beta (TGF-β). There remains uncertainty on how to interpret biomarker results, and significance in levels [32]. Due to this ambiguity, biomarkers were not included in the Index in this study.

## Discussion

The Index developed here is a collection of existing testing methods already utilised in published studies. There are likely other assessments that are relevant, but have not been included here. The Index is a framework, the first step towards a unified assessment of mesh tissue integration in the abdominal wall, with the hopes that there could be an objective reference of mesh performances under various conditions to which surgeons may refer to. The Index is applicable to any mesh or mesh-like implant in any layer in the abdominal wall. Adhesion score is only relevant for intraperitoneal placement, and is not calculated for non-intraperitoneal positions. The mathematics involved here has been kept relatively simple, such that only averaging of relevant data needs to be performed. This allows easy modification in future iterations of the Index if new problems are identified, or if the initial premise is proven wrong.

Mesh tissue integration is not a new concept. Literature descriptions of tissue incorporation into synthetic mesh for hernia repair can be found as early as 1983 [33]. Importance of tissue infiltration and the effect of textile porosity was highlighted by Mühl et al.’s work on effective porosity [8]. Cobb et al. used tissue infiltration as one of the main arguments for the use of lightweight macroporous mesh, during a time when heavyweight microporous mesh was still being used [34]. With increasing recognition of the relevance of tissue integration, manufacturers began to include this within their marketing materials, often backed by findings from animal studies. Numerous studies published over the recent decades have included tissue integration and fibrosis as part of their assessment of the mesh tissue interface. While there have been limited comparison of performance between mesh products within a single study, there has been very little standardisation among methodology and animal models used [14, 35].

Comparing results across animal studies investigating mesh tissue integration has been difficult. Some authors have attempted to unify animal study protocols [36], though largely unsuccessful, likely due to differences in budget, facility limitations, variations in animal supply and difficulty replicating experimental conditions. Without a solution to these problems, the focus shifted towards establishing a unified system to grade mesh products [6]. Using a unified scoring system, differences among methodology and animal models are accommodated and data may be interpreted on the same platform. The pilot study showed that Index scores change over time with mesh following implantation, and a singular score is likely not possible to be assigned to mesh products, as mesh products may have different behaviours over time [11].

The Index, by design, only attempts to quantify the changes at the mesh tissue interface, and expresses this as a numerical value. While this design avoids several problems identified in the pilot study, it inherently does not contain any means for interpretation and as such is not directly applicable to clinical practice, without correlating with clinical outcomes. The Index requires dedicated animal studies to investigate the mesh tissue interface. Clinicians are not expected to calculate the Index during their clinical practice, as much of the information required will be laboratory findings.

As with any systems invention, troubleshooting of problems and system bugs is required before full deployment and usage. The current iteration of the Index will need to be tested with living tissue to ensure it functions as intended, by demonstrating numerical and statistically significant changes among its four domains over time, and be in keeping with known clinical observations.

Optimal test conditions is to use fresh explanted mesh tissue specimens from living abdominal walls over multiple time points. As some of the assessments have been pooled from multiple studies with new parameters, there is likely no single existing study that has data that can be utilised fully to validate the Index. The invasiveness of tissue sampling is not practical in humans as a clinical trial, and existing explant tissue banks can only provide partial validation due to interference from formalin fixation. Cadaveric human models do not allow mesh to be integrated. Organoids are not yet sufficiently developed to replicate the complexity of the abdominal wall [37]. Animal models are likely the most accessible and feasible option to validate the Index.

Recognising that animal models have limitations in translating results to clinical practice, the Index is best served by being coupled with a hernia registry. A learning healthcare system clinical quality registry will allow tracking of long-term outcomes in hernia repairs, and record the behaviour of mesh products utilised. This could be mandated at a surgeon level and legislated at a national level as part of annual accreditation, which could improve the transparency of hernia mesh medical device regulations.

The inclusion of the Index in a clinical quality registry forms a benchtop to bedside workflow, and potentially facilitates identification of adjunct treatments for specific patient groups. For example, a mesh identified by animal studies to have optimal integration and minimal fibrosis may still perform poorly in comorbid patients with diabetes or immunosuppression. In such cases, the addition of adjuncts, such as platelet rich fibrin or other therapeutic agents, may be found to have a beneficial influence on their Index scores.

In conclusion, a Mesh Integration (MINT) Index has been developed for standardised assessment of in vivo hernia mesh behaviour. Validation studies using a porcine model with placement of synthetic mesh to the retrorectus and intraperitoneal layer have been preregistered by the authors and are underway (Digital Object Identifier [DOI] 10.17590/asr.0000350 and 10.17590/asr.0000371).

## Supplementary Information

Below is the link to the electronic supplementary material.

Supplementary file1 (DOCX 73 KB)

Supplementary file2 (DOCX 74 KB)

Supplementary file3 (DOCX 78 KB)

## Data Availability

Data have been provided in manuscript and supplementary.

## Abbreviations

ATR-FTIR: Attenuated total reflectance Fourier’s transformation infrared spectroscopy
ATSM: American Society for Testing and Materials International
FDA: Food and Drug Administration (of the United States)
ISO: International Organization for Standardization
META: Mesh tissue adhesion score
MMP: Matrix metalloproteinase
SEM: Scanning electron microscopy
TGF-β: Transforming growth factor beta
UoA: University of Adelaide
VEGF: Vascular endothelial growth factor
