# Supplementary material for: Introducing the Mesh Integration (MINT) Index: a standardised ratio scale for assessing in vivo hernia mesh performance
Source: Surg Endosc. 2025 Sep 2;39(10):7052–63. doi: 10.1007/s00464-025-12098-1 (PMC12500770; doi:10.1007/s00464-025-12098-1)
Supplement: Supplementary file 1 — Supplementary file1 (DOCX 73 KB) [file 464_2025_12098_MOESM1_ESM.docx]

**Supplementary 1 –** Visual degradation scoring worksheet, adapted from Krieg et al. 2021.

**Degradation Scoring Worksheet**

| Tissue Sample Serial No. : | Date of assessment:  Assessor: |
| --- | --- |

Instructions: Mark the corresponding column in each row based on identification

| **Observation** | **Score 0** | **Score 1** | **Score 2** |
| --- | --- | --- | --- |
| **Surface changes observed with scanning electron microscopy, magnification x1000** | No change / not applicable | Coating / non-mesh component only | Mesh involved |
| **Surface changes observed with light microscopy, magnification x10** | No change / not applicable | Coating / non-mesh component only | Mesh involved |
| **Surface changes observed with unaided vision** | No change / not applicable | Coating / non-mesh component only | Mesh involved |
| **Loss of integrity when handled gently with fine non-toothed surgical forceps** | No change / not applicable | Coating / non-mesh component only | Mesh involved |

Surface changes: brittleness, crack, tear, crazing, hardening, loose, peel, break

Loss of integrity: abrasion, chip, flaking, loss of material, crumbling, scratch

Comments:

References:

Krieg T, Mazzon C, Gómez-Sánchez E. Material Analysis and a Visual Guide of Degradation Phenomena in Historical Synthetic Polymers as Tools to Follow Ageing Processes in Industrial Heritage Collections. Polymers (Basel). 2021 Dec 29;14(1).
