# Supplementary material for: Introducing the Mesh Integration (MINT) Index: a standardised ratio scale for assessing in vivo hernia mesh performance
Source: Surg Endosc. 2025 Sep 2;39(10):7052–63. doi: 10.1007/s00464-025-12098-1 (PMC12500770; doi:10.1007/s00464-025-12098-1)
Supplement: Supplementary file 2 — Supplementary file2 (DOCX 74 KB) [file 464_2025_12098_MOESM2_ESM.docx]

**Supplementary 2 –** Visual adhesion scoring worksheet, adapted from van der Hill et al. 2020.

**Adhesion Scoring Worksheet**

| Tissue Sample Serial No. : | Date of assessment:  Assessor: |
| --- | --- |

Instructions: Mark the corresponding column in each row based on identification

| **Observation** | **Score 0** | **Score 1** | **Score 2** | **Score 3** | **Score 4** |
| --- | --- | --- | --- | --- | --- |
| **Percentage of mesh surface covered with adhesions** | 0% | 1-25% | 26-50% | 51-75% | 76-100% |
| **Tenacity** | No adhesions | Loose adhesions easily released by traction only | Adhesions require sharp dissection, no organ/serosal damage | Adhesions require sharp dissection, with unavoidable organ/serosal damage |  |
| **Thickness of adhesions** | No adhesions | Single thin, filmy adhesions | Multiple thin, filmy adhesions | Single dense adhesion with or without filmy adhesions | Multiple dense adhesions with or without filmy adhesions |
| **Organ involvement** | No adhesions | Adhesions between mesh and omentum or a solid organ | Adhesions between mesh and part(s) of the intestinal tract | Adhesions between mesh and part(s) of the intestinal tract with enteric fistulas or bowel erosions |  |

*Scoring system adapted from META score by van den Hil et al.*

Comments:

References:

van den Hil LCL, Mommers EHH, Bosmans JWAM, et al. META Score: An International Consensus Scoring System on Mesh-Tissue Adhesions. World Journal of Surgery. 2020;44(9):1
