# Supplementary material for: Introducing the Mesh Integration (MINT) Index: a standardised ratio scale for assessing in vivo hernia mesh performance
Source: Surg Endosc. 2025 Sep 2;39(10):7052–63. doi: 10.1007/s00464-025-12098-1 (PMC12500770; doi:10.1007/s00464-025-12098-1)
Supplement: Supplementary file 3 — Supplementary file3 (DOCX 78 KB) [file 464_2025_12098_MOESM3_ESM.docx]

**Supplementary 3 –** Visual histology scoring worksheets. Adapted from ISO 10993-6:2016, Keating et al 2019, and Jenkins et al 2011.

**Histology Scoring Worksheet**

| Tissue Sample Serial No. : | Date of assessment:  Assessor: |
| --- | --- |

Instructions: Mark the corresponding column in each row based on identification

PART 1 of 2

| **Observation** | **Score 0** | **Score 1** | **Score 2** | **Score 3** | **Score 4** |
| --- | --- | --- | --- | --- | --- |
| **Polymorphonuclear cells** | Absent | Rare, 1-5 per high powered field | 5-10 per high powered field | Heavy infiltrate | Packed |
| **Lymphocytes** | Absent | Rare, 1-5 per high powered field | 5-10 per high powered field | Heavy infiltrate | Packed |
| **Plasma cells** | Absent | Rare, 1-5 per high powered field | 5-10 per high powered field | Heavy infiltrate | Packed |
| **Macrophages** | Absent | Rare, 1-5 per high powered field | 5-10 per high powered field | Heavy infiltrate | Packed |
| **Giant cells** | Absent | Rare, 1-5 per high powered field | 5-10 per high powered field | Heavy infiltrate | Sheets |
| **Cellular infiltration** | Absent | Cells present at implant periphery, no penetration into scaffold | Cells infiltrate implant but do not reach centre | Cells penetrate into centre of implant | Cells diffusely expand and distort implant |
| **Neovascularisation** | Absent | Minimal capillary proliferation, focal, 1-3 buds | Groups of 4-7 capillaries with supporting fibroblastic structures | Broad band of capillaries with supporting structures | Extensive band of capillaries with supporting fibroblastic structures |
| **Connective tissue deposition** | Absent | Restricted to periphery of implant | Present within implant interstitium, not extending into centre | Present throughout implant, including centre | Diffusely expands and distorts implant |

*High powered field: 40× object*

*Scoring system adapted from ISO 10993-6:2016, Keating et al & Jenkins et al.*

**Please turn over. →**

Instructions: Mark the corresponding column in each row based on identification

PART 2 of 2

| **Observation** | **Score 0** | **Score 1** | **Score 2** | **Score 3** | **Score 4** |
| --- | --- | --- | --- | --- | --- |
| **Fibrosis** | Absent | Narrow band, 1-2 cell layers thick | Moderately thick band, <10 cell layers thick | Thick band, contiguous band along length of tissue | Extensive band, thick zone with effacement of local architecture |
| **Fatty infiltration** | Absent | Minimal amount of fat associated with fibrosis | Several layers of fat and fibrosis | Elongated and broad accumulation of fat cells about the implant site | Extensive fat completely surrounding the implant |
| **Fibrous encapsulation** | Absent | Minimal, 1-25% of periphery | Mild, 26-50% of periphery | Moderate, 51-75% of periphery | Extensive encapsulation, 76-100% |
| **Mineralization** | Absent | Minimal, focal, nearly imperceptible | Mild, focally extensive, inconspicuous | Moderate, multifocal, locally extensive, readily apparent | Severe, regionally extensive, overwhelming with effacement of regional architecture |
| **Implant degradation** | Absent, implant intact, borders clearly demarcated | Implant minimally degraded, <10%, with some separation by host tissue/infiltrates | Implant notably degraded, ~10-50%, difficult to distinguish scaffold from host tissue | Implant markedly degraded, >50%, difficult to distinguish scaffold from host tissue | No evidence of implant remaining |
| **Necrosis** | Absent | Minimal, focal, nearly imperceptible | Mild, focally extensive, inconspicuous | Moderate, multifocal, locally extensive, readily apparent | Severe, regionally extensive, overwhelming with effacement of regional architecture |

*High powered field: 40× object*

*Scoring system adapted from ISO 10993-6:2016, Keating et al & Jenkins et al.*

Comments:

References:

Jenkins ED, Melman L, Deeken CR, Greco SC, Frisella MM, Matthews BD. Evaluation of fenestrated and non-fenestrated biologic grafts in a porcine model of mature ventral incisional hernia repair. Hernia. 2010 2010-12;14(6):599-610.

Keating JH, Melidone R, Garcia-Polite F. Preclinical Evaluation of Mesh Implants: The Pathologist’s Perspective. Toxicologic Pathology. 2019;47(3):379-89.
